# Atrial Lesions in a Pedigree With *PRKAG2* Cardiomyopathy: Involvement of Disrupted AMP-Activated Protein Kinase Signaling

**DOI:** 10.3389/fcvm.2022.840337

**Published:** 2022-03-10

**Authors:** Shaojie Chen, Yongping Lin, Yue Zhu, Le Geng, Chang Cui, Zhaomin Li, Hailei Liu, Hongwu Chen, Weizhu Ju, Minglong Chen

**Affiliations:** ^1^Division of Cardiology, The First Affiliated Hospital of Nanjing Medical University, Nanjing, China; ^2^Department of Cardio-Thoracic Surgery, The First Affiliated Hospital of Nanjing Medical University, Nanjing, China

**Keywords:** *PRKAG2*, pedigree, atrial arrhythmia, histology, glycogen deposition

## Abstract

PRKAG2 cardiomyopathy is a rare progressive disease characterized by increased ventricular wall thickness and preexcitation. Dysfunction of the protein 5′-AMP-activated protein kinase (AMPK) plays a decisive role in the progression of ventricular lesions. Although patients with the *PRKAG2*-R302Q mutation have a high incidence of atrial fibrillation (AF), the molecular mechanism contributing to the disease remains unclear. We carried out whole-genome sequencing with linkage analysis in three affected members of a family. Atrial samples were obtained from the proband *via* surgical intervention. Control atrium biopsies were obtained from patients with persistent AF. Pathological changes were analyzed using the hematoxylin and eosin (H&E), Masson, and periodic acid–Schiff (PAS) staining. The AMPK signaling pathway was investigated by western blot. A murine atrial cardiomyocyte cell line (HL-1) and human induced pluripotent stem derived atrial cardiomyocytes (hiPSC-ACMs) were transfected with an adenovirus carrying the same mutation. We used enzyme linked immunosorbent assay (ELISA) to determine the AMPK activity in HL-1 cells and hiPSC-ACMs overexpressing *PRKAG2*-R302Q. Pathological results showed a large quantity of glycogen accumulation and vacuolization in cardiomyocytes from the proband atrial tissue. Western blot analysis revealed that the AMPK activity was significantly downregulated compared with that of the controls. Furthermore, remarkable glycogen deposition and impairment of AMPK activity were reproduced in HL-1 cells overexpressing *PRKAG2*-R302Q. Taken together, *PRKAG2*-R302Q mutation directly impair atrial cardiomyocytes. *PRKAG2*-R302Q mutation lead to glycogen deposition and promote the growth of atrial lesions by disrupting the AMPK pathway.

## Introduction

The *PRKAG2* gene was mapped to chromosome 7, encoding the γ2 subunit of AMP-activated protein kinase (AMPK) (1). AMPK occurs as a heterotrimeric kinase comprising 1 catalytic (α) and 2 regulatory (β and γ) subunits. The γ2 subunit, along with γ1 and γ3, has four individually conserved cystathionine β-synthase (CBS) domains, each of which can bind one molecule of AMP or ATP (2). Different ratios of AMP/ATP binding to the γ2 subunit result in different levels of AMPK activity. When activated, AMPK accelerates glycolysis and suppresses inflammatory and endoplasmic reticulum stress (3–5), exerting cardioprotective effects.

Cardiomyopathy caused by *PRKAG2* gene mutation is called *PRKAG2* cardiomyopathy. *PRKAG2* cardiomyopathy is usually associated with ventricular hypertrophy, conduction disease, and progressive glycogen storage (6, 7). Familial ventricular preexcitation in *PRKAG2* cardiomyopathy is thought to be associated with Mahaim fibers, which have atrioventricular node-like conduction properties (8). *PRKAG2* mutations are autosomal dominant and tend to affect several family members of one pedigree. Increased activity of the insulin pathway, a widely accepted hypertrophic signal, was found to be responsible for cardiomyocyte hypertrophy caused by the *PRKAG2* gene mutation (9).

Recently, a high incidence of atrial fibrillation was reported for patients with the *PRKAG2*-R302Q mutation (10). However, few of the molecular mechanisms involved in this process have been explored. The role of the AMPK pathway in *PRKAG2* cardiomyopathy carrying the R302Q mutation remains controversial. Patient-specific induced pluripotent stem cell (iPSC)-derived cardiomyocytes have exhibited increased AMPK activities (11, 12). Acute adenoviral-mediated expression of the γ2 mutant (R302Q) in isolated cardiomyocytes resulted in a significant increase in AMPK activity (13). A transgenic mouse model demonstrated reduced AMPK activity (14). Although patient-specific iPSC-CMs harbor the same gene background as the host, these cells are immature compared to adult cardiomyocytes, including in terms of metabolic properties. Hence, pathological evidence from patient biopsies would be extremely useful for understanding the underlying mechanisms.

We clinically identified a Chinese pedigree with the *PRKAG2* p. R302Q mutation. The mutation carriers showed histories of syncope, atrial tachycardia, embolic events and pacemaker implantation. We obtained atrial samples from the proband *via* surgical intervention and compared the histological and molecular characteristics to those of biopsies of AF patients undergoing cardiopulmonary bypass procedures. We verified the pathogenicity of the *PRKAG2*-R302Q mutation using two kinds of atrial muscle cells overexpressing *PRKAG2*-R302Q. Our results provide solid pathological evidence of atrial lesions caused by the *PRKAG2*-R302Q mutation, as well as evidence of an impaired AMPK protein activation mode in response to different stimuli, shedding light on potential therapeutic strategies for atrial cardiomyopathy.

## Materials and Methods

### Research Subjects

Patient samples, including venous blood samples and left atrial appendage, were taken after obtaining informed consent. This study was approved by the Scientific Ethics committee of the First Affiliated Hospital of Nanjing Medical University (2014-SR-090). The detailed clinical assessments were included in the Supplementary Methods.

### Whole-Exome Sequencing and Sanger Sequencing

Peripheral blood samples were obtained from the peripheral venous blood of five family members (II-1, II-3, II-5, III-1, and III-2). DNA was extracted using a QIAamp DNA Blood Maxi Kit. A TruSeq™ Exome Enrichment Kit (Illumina, San Diego, CA, United States) was used to capture exomes. The DNA library was quantified using the Quant-iT™ PicoGreen^®^ dsDNA Assay Kit (LIFE). Sanger sequencing was performed to validate the mutation.

### Functional Assays

The experimental details, including cell culture, transfection, staining, western blot, and ELISA are available in Supplementary Material.

### Statistical Analysis

The difference between the two groups was assessed using Student’s *t*-test. The statistical analysis was performed using SPSS Statistics 19.0 (IBM, Chicago, IL, United States). A *p*-value < 0.05 was considered statistically significant.

## Results

### Characteristics of a Patient Carrying the *PRKAG2*-R302Q Mutation

**FIGURE 1:**
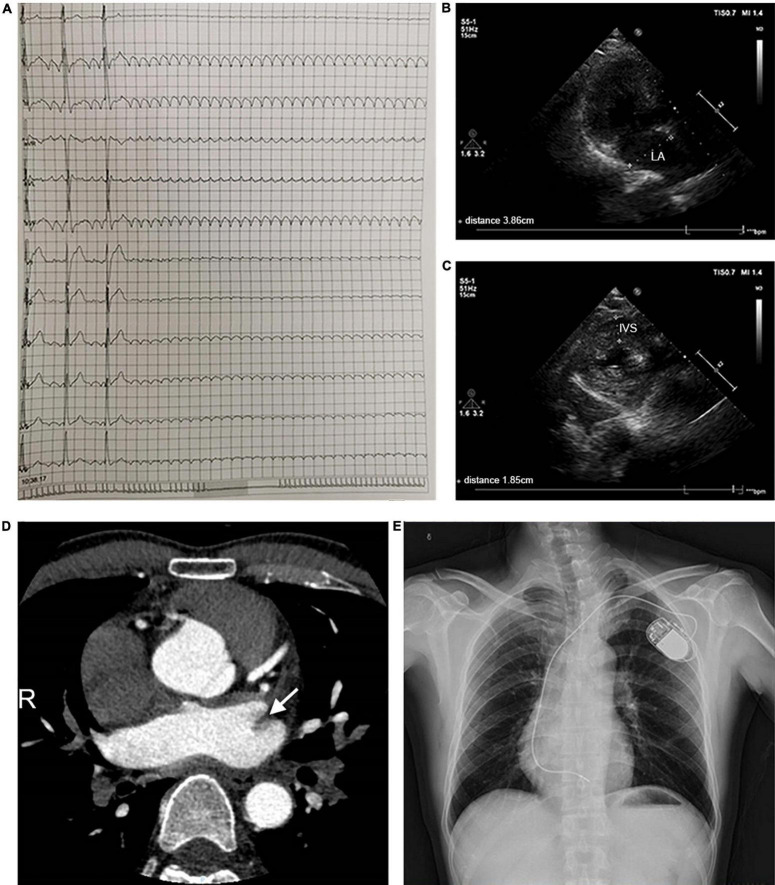
Clinical parameters for the pedigree. **(A)** 24-h Holter monitoring of the proband. **(B,C)** Echocardiographic parameters, including LAD and IVS. **(D)** The cardiac CT-scan indicated atrial thrombus (shown by the arrow). **(E)** A posteroanterior chest X-ray was taken immediately after pacemaker implantation. Abbreviations: LA, left atrium; IVS, interventricular septum.

A 44-year-old patient from a family with suspected inherited cardiomyopathy was hospitalized for the presentation of palpitations and syncope. Atrial flutter and intermittent high-grade AV block (the longest RR was 11.275 s) were observed under 24-h Holter-ECG monitoring (Figure 1A). An electrocardiogram (ECG) of the proband simultaneously revealed typical atrial flutter (Supplementary Figure 1A). Echocardiography demonstrated that the proband’s atrium was enlarged [left atrium diameter (LAD): 39 mm (Figure 1B); right atrium diameter (RAD): 38 mm], whereas both the ejection fraction (EF) and left ventricular end-diastolic diameter (LVDd) were normal (Supplementary Table 1). The proband’s interventricular septum was hypertrophic (IVS: 18 mm, Figure 1C), but neither ST-segment change nor T-wave inversion was observed in the ECG. Considering that left atrial hemodynamics can be altered by atrial flutter, the left atrial appendage (LAA) was analyzed in detail using enhanced cardiac CT scans. The results indicated the presence of an LAA thrombus (Figure 1D). Considering the proband’s diseases and probable thrombus formation, radiofrequency ablation of atrial flutter was terminated. A permanent pacemaker was implanted instead. After implantation, a posteroanterior chest X-ray showed no evident abnormality in the size and shape of the ventricle (Figure 1E).

### Pedigree Investigation

**FIGURE 2:**
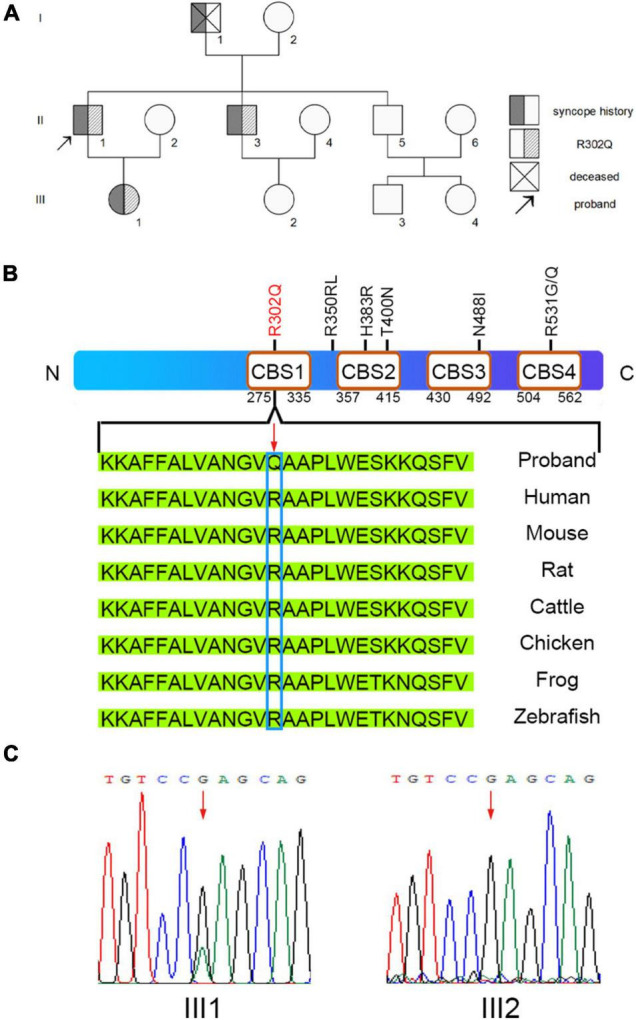
Sequence analysis for the pedigree. **(A)** Overview of the clinical symptoms for the pedigree. The proband is indicated by the black arrow. **(B)** The missense mutation *PRKAG2*-R302Q was found in the proband. **(C)** A heterozygous G > A transition at nucleotide 905 of *PRKAG2* was confirmed by Sanger sequencing. A change in the amino acid sequence is highlighted by the red arrow.

A pedigree investigation demonstrated that family members I-1, II-3, and III-1, in addition to the proband (II-1), complained of a history of syncope (Figure 2A). II-3 was previously diagnosed with atrial flutter and hypertrophic cardiomyopathy (Supplementary Table 1) and was implanted with a pacemaker. The ECG data of III-1 showed sinus rhythm with slight, short PR intervals but did not meet the criteria for preexcitation. There was no evidence of structural heart defects in the echocardiogram of III-1 (Supplementary Table 1).

We collected the peripheral blood of the family members for whole-exome sequencing (WES). WES analysis identified a heterozygous, missense mutation c.905G > A (R302Q) in the *PRKAG2* genes of II-1 and II-3 (Figure 2B). Further Sanger sequencing confirmed that III-1 was a *PRKAG2*-R302Q mutation heterozygous carrier, whereas III-2 was a negative non-carrier (Figure 2C). Given that *PRKAG2* syndrome is usually associated with early-onset ventricular preexcitation, a long-term follow-up of III-1 was recommended.

### Pathological and Molecular Profiles of the Atria With the *PRKAG2*-R302Q Mutation

After the proband had received 4 months of anticoagulation therapy, the CT scan data still indicated a possible thrombus in the LAA (Supplementary Figure 1B). Thus, left atrial appendectomy was performed for stroke prevention, together with surgical ablation of the tricuspid isthmus. We then compared the proband’s samples to LAA tissues from two age-matched individuals with AF undergoing surgical interventions (Supplementary Figure 2 and Supplementary Table 1).

**FIGURE 3:**
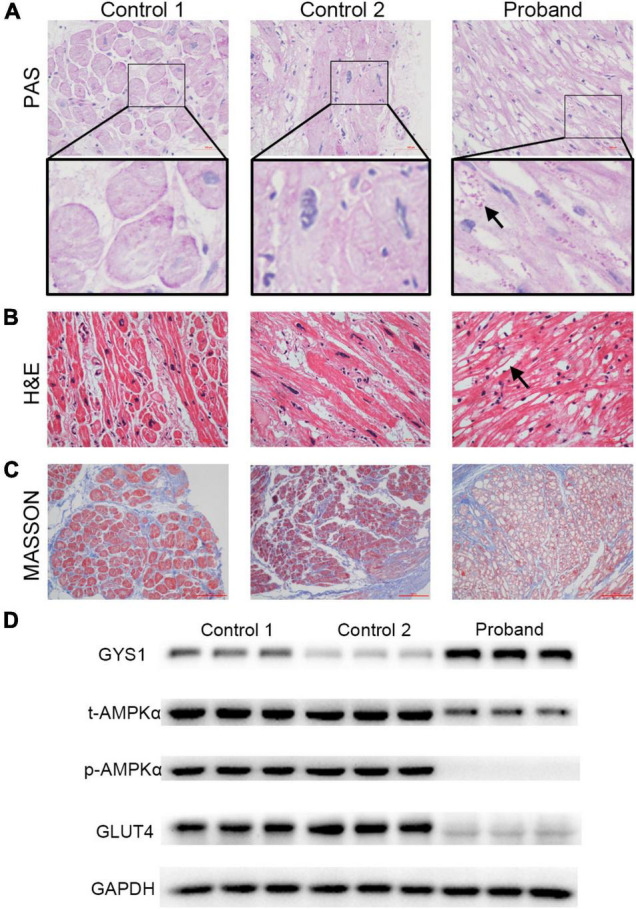
Alterations in the pathology and protein expression of the left atrial appendage tissues of the proband. **(A)** Representative photograph of PAS staining results. Many glycogen granules were observed (indicated by the black arrow, scale bar = 100 μm). **(B)** Representative images of H&E staining results. Distinct vacuolated myocytes (indicated by the black arrow) are visible (scale bar = 50 μm). **(C)** Observation of severe fibrosis in cardiac tissues by Masson staining (scale bar = 50 μm). **(D)** Western blotting of GYS1, t-AMPKα, p-AMPKα, GLUT4, and GAPDH. Abbreviations: GYS1, glycogen synthase 1; AMPK, 5′-AMP-activated protein kinase; GLUT4, glucose transporter 4.

Pathological changes, including glycogen storage and fibrosis, are one of the specific manifestations of myocardial lesions caused by the *PRKAG2*-R302Q mutation. In Periodic Acid-Schiff (PAS) stained sections of the LAA of the proband, large numbers of glycogen granules were observed in the mutant cardiomyocytes, whereas there was no evidence of gross glycogen deposition in the controls (Figure 3A). Unlike the controls, intense vacuolization of cardiomyocytes from the proband was observed by the H&E staining (Figure 3B). This could be generated by the presence of glycogen deposits, which were not visible in the H&E staining. Prominent blue-stained collagen fibers from Masson’s staining were observed in all three samples (Figure 3C).

To gain insights into the molecular basis for the *PRKAG2*-R302Q mutation, AMPK activity was assessed by western blotting (Figure 3D). Total AMPKα (t-AMPKα) was weakly expressed in the mutant atrial myocardium. Subsequently, phosphorylation of AMPKα (p-AMPKα) expression was barely detectable. Consistent with the PAS staining results, expression of robust glycogen synthase 1 (GYS1), a rate-limiting enzyme of the glycogen synthesis pathway, was observed in the proband’s atrial tissue, unlike in the controls, indicating enhanced glycogen synthase activity. By contrast, glucose transporter 4 (GLUT4), a widely recognized downstream protein for AMPK, was highly expressed in two controls.

### Atrial Cardiomyocytes Overexpressing *PRKAG2*-R302Q Replicated Lesions

**FIGURE 4:**
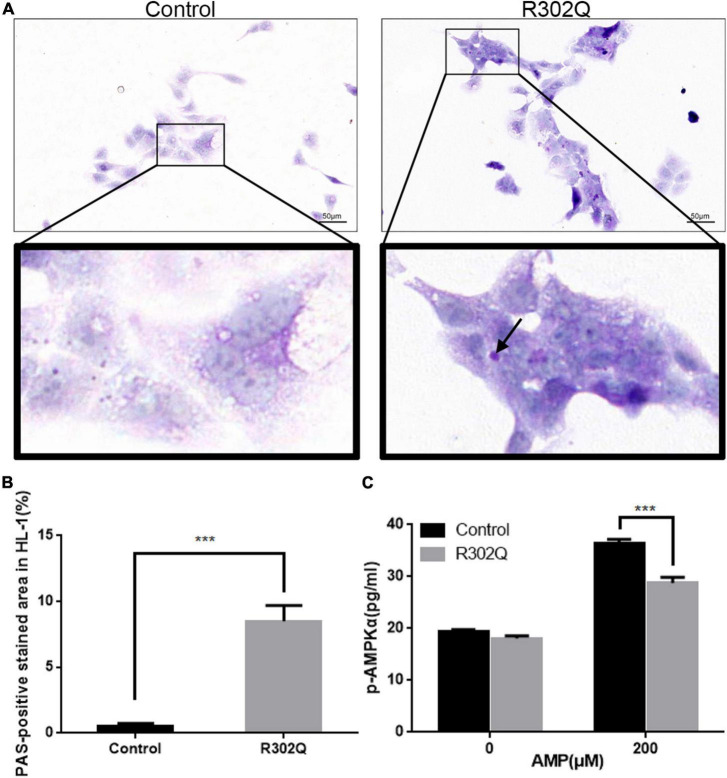
Molecular changes in HL-1 cells overexpressing *PRKAG2*-R302Q. **(A)** The glycogen content in HL-1 cells was analyzed by PAS staining (scale bar = 50 μm). **(B)** Quantitative analysis of the PAS-positive stained area in HL-1 cells. **(C)** The level of p-AMPKα expression was determined by ELISA (*n* = 3). All data were analyzed by the Student’s *t*–test (*n* = 3, ***p < 0.001).

The HL-1 cell line was transfected with an adenovirus carrying the *PRKAG2*-R302Q mutation. The results demonstrated that overexpressing the *PRKAG2*-R302Q mutation in HL-1 murine atrial cardiomyocytes resulted in PAS-positive glycogen deposition compared with the vector control (Figures 4A,B).

A quantitative ELISA for the phosphorylation of AMPKα was used to evaluate AMPK activity under different conditions. At baseline, there were no differences in the p-AMPKα concentration of the mutant and control groups in HL-1 cells. Under stimulation with 200 μM AMP, both groups exhibited increased AMPK activity. However, the p-AMPKα concentration in the *PRKAG2*-R302Q group was considerably lower than that in the control group (Figure 4C). Moreover, hiPSC-ACMs overexpressing *PRKAG2*-R302Q replicated the similar outcomes (Supplementary Figure 5).

### Characteristics of the Proband and II-3 at the 3-Year Follow-Up

At the 3-year follow-up, the proband and II-3 underwent ECG and echocardiography examinations. The ECG showed that II-3 suffered from atrial flutter, whereas the proband maintained sinus rhythm (Supplementary Figure 3). However, atrial arrhythmia episodes were recorded in both patients using 24-h Holter-ECG monitoring (Supplementary Figure 4). Echocardiography suggested that both patients exhibited enlarged atriums and increased interventricular septum thicknesses (Supplementary Table 2).

## Discussion

Clinically, *PRKAG2* mutations cause glycogen-storage cardiomyopathy, ventricular preexcitation, and conduction system degeneration (15, 16). Typical clinical symptoms can develop at approximately 40 years. In 70% of cases, ventricular preexcitation is the first symptom of *PRKAG2* cardiomyopathy (17). To date, several mutation sites of the *PRKAG2* gene have been confirmed to be pathogenic, among which R302Q is the most common mutation (18).

A study has suggested that patients from a pedigree carrying the *PRKAG2*-R302Q mutation all showed left atrial enlargement, but some patients were diagnosed with no or mild ventricular hypertrophy (10). Thus, atrial impairments might occur in the early stage of *PRKAG2* cardiomyopathy. In our present study, the 44-year-old proband and his younger brother were diagnosed with atrial flutter and implanted with pacemakers. Masson and H&E staining revealed fibrosis and vacuolization of the proband’s atrial tissue, suggesting severe atrial damage.

The *PRKAG2* gene encodes the γ2 subunit of AMPK. AMPK is highly expressed in human cardiac muscle and plays a vital role in regulating cellular energy homeostasis and metabolism (19). In a hypertrophic cardiomyopathy (HCM) mouse model expressing mutant α*-MyHC*, activation of AMPK was limited. As a result, lipid uptake and the cardiomyocyte content were reduced, and ventricular systolic function was impaired (20). After AMPK was effectively activated, the phenotype of ventricular dysfunction could be restored. Patient-specific iPSCs carrying *PRKAG2* mutation-derived cardiomyocytes exhibited increased AMPK activities (11, 12). Acute adenoviral-mediated expression of the γ2 mutant (R302Q) in isolated cardiomyocytes resulted in a significant increase in AMPK activity (13). In this study, western blot analysis was performed on the atrial tissue of a proband carrying the *PRKAG2*-R302Q mutation, and both the p-AMPK/t-AMPK ratio and t-AMPK content decreased significantly. Our result was consistent with studies on a transgenic mouse model (14, 21). Overall, the *PRKAG2*-R302Q mutation can directly impair the atrium by disrupting AMPK signaling.

Each γ subunit of AMPK combine with four molecules of AMP and ATP. Thus, the higher the quantity of AMPK bound to AMP is, the higher the activation of AMPK is. Free AMP had a low content in the myocardium but a higher affinity for AMPK than ATP (22). However, an essential feature of the *PRKAG2*-R302Q mutation is the significantly reduced affinity between AMP and the γ2 subunit of AMPK, which prevents effective activation of AMPK. In the present study, AMPK activation was limited in the mutant group compared with the control group in response to high concentration of AMP. These results confirmed that AMPK activity was suppressed in patients with the *PRKAG2*-R302Q mutation in response to endogenous or exogenous stimuli. Thus, cardiac function could not be effectively preserved.

On the other hand, cardiac glucose uptake is primarily mediated by glucose transporter 4 (GLUT4), which is found to be regulated by AMPK. Previous studies confirmed that expression of GLUT4 was enhanced by upregulated AMPK activity (23, 24) and understandably limited by disrupted activation of AMPK. Moreover, GYS1 is identified as a key enzyme involved in glycogen synthesis. In a transgenic mice model harboring a knock-in mutation in GYS1 and mutation of the *PRKAG2*, the GYS1 activity was significantly reduced and glycogen accumulation was markedly eliminated (25). All these evidences suggested that glycogen deposits in *PRKAG2* cardiomyopathy was due to enhanced expression of GYS1. In addition, a large accumulation of glycogen has been reported to cause preexcitation by damaging annulus fibrosis at the atrioventricular connection (25, 26). Using PAS staining, we also found a large accumulation of glycogen in the proband’s atrial tissue and HL-1 cells overexpressing *PRKAG2*-R302Q, which may also be a substrate of arrhythmia.

In the clinical view, the present study elucidated the mechanism of *PRKAG2*-R302Q mutation leading to atrial lesions. Clinicians were also reminded to pay attention to the possibility of early onset of atrial lesions in addition to ventricular lesions caused by *PRKAG2* cardiomyopathy, and timely intervention treatment.

## Study Limitation

First, only one cell line was used in this study. However, HL-1 cell line, derived from mouse atrial myocytes, has been widely used to explore cardiac morphological, biochemical, and electrophysiological properties. The results are representative and stable. Moreover, hiPSC-ACM, though immature, overexpressing *PRKAG2*-R302Q mutation could well mimic the properties of atrial cardiomyocytes of the patients. Second, not all the family member underwent WES or Sanger sequencing. Their gene backgrounds need further identification. Third, under the condition of disrupted AMPK signaling due to *PRKAG2*-R302Q mutation, the mechanism of atrial myocytes being more vulnerable to stimuli needs to be further investigated.

## Conclusion

In the present study, we obtained solid evidence that the *PRKAG2*-R302Q mutation directly caused atrial lesions. Impaired AMPK activation is the underlying pathogenic mechanism. These results are useful for the early diagnosis and intervention of patients with *PRKAG2* cardiomyopathy.

## Data Availability Statement

The datasets presented in this study can be found in online repositories. The names of the repository/repositories and accession number(s) can be found below: https://www.ncbi.nlm.nih.gov/, SRR17257561; https://www.ncbi.nlm.nih.gov/, SRR17257560; and https://www.ncbi.nlm.nih.gov/, SRR17257559.

## Ethics Statement

The studies involving human participants were reviewed and approved by the Bioethics Committee of the First Affiliated Hospital of Nanjing Medical University. Written informed consent to participate in this study was provided by the participants’ legal guardian/next of kin. Written informed consent was obtained from the individual(s) for the publication of any potentially identifiable images or data included in this article.

## Author Contributions

SC, YL, and YZ performed the experiments, analyzed the data, and drafted the manuscript. LG contributed to the acquisition of human tissue samples. CC made contributions to the conception, design of the study, and revision of the manuscript. ZL, HL, and HC were involved in revising the manuscript. MC and WJ contributed to the guidance on the whole study. All authors contributed to the article and approved the submitted version.

## Conflict of Interest

The authors declare that the research was conducted in the absence of any commercial or financial relationships that could be construed as a potential conflict of interest.

## Publisher’s Note

All claims expressed in this article are solely those of the authors and do not necessarily represent those of their affiliated organizations, or those of the publisher, the editors and the reviewers. Any product that may be evaluated in this article, or claim that may be made by its manufacturer, is not guaranteed or endorsed by the publisher.

## Abbreviations

AMPK: AMP-activated protein kinase
CBS: cystathionine β -synthase
ECG: electrocardiogram
LAD: left atrium diameter
RAD: right atrium diameter
EF: ejection fraction
LVDd: left ventricular end-diastolic diameter
IVS: interventricular septum
LAA: left atrial appendage
WES: whole-exome sequencing
HCM: hypertrophic cardiomyopathy
iPSC: induced pluripotent stem cell
GYS1: glycogen synthase 1
GLUT4: glucose transporter 4.
