# Supplementary material for: Atrial Lesions in a Pedigree With PRKAG2 Cardiomyopathy: Involvement of Disrupted AMP-Activated Protein Kinase Signaling
Source: Front Cardiovasc Med. 2022 Mar 10;9:840337. doi: 10.3389/fcvm.2022.840337 (PMC8960295; doi:10.3389/fcvm.2022.840337)
Supplement: Supplementary file 1 [file Data_Sheet_1.pdf]

Supplementary Materials

The supplementary materials included Supplementary methods, Supplementary Figure1-5, and Supplementary Table 1-2.

Supplementary methods

1. ECG and 24-h Holter

All patients received a standard 12-lead electrocardiography (ECG) at rest (10 mm/mV, 25 mm/s). The proband and II-3 underwent 24-h Holter monitoring to investigate the causes of palpitations and syncope.

2. Echocardiographic parameters

Cardiac function was evaluated by echocardiography. Ultrasound measurements included the interventricular septal thickness (IVS), left atrium diameter (LAD), right atrium diameter (RAD), ejection fraction (EF), etc. We collected echocardiographic data from the proband (II-1), his daughter (III-1) and younger brother (II-3) upon diagnosis of the *PRKAG2*-R302Q mutation. Echocardiographic data were also obtained from two control patients.

3. CT imaging

Preoperative evaluation of the left atrial appendage was performed by cardiac computed tomography (CT) to rule out thrombus formation.

4. Chest X-ray

Postoperative, posteroanterior chest X-rays were taken to verify the pacemaker location and determine the size of the heart.

5. Cell culture and cell transfection

A murine atrial cardiomyocyte cell line (HL-1) was cultured in Claycomb medium (Sigma-Aldrich) containing 10% fetal bovine serum, 100 U/ml penicillin/streptomycin, 0.1 mM norepinephrine, and 2 mM l-glutamine (Sigma–Aldrich).
hiPSCs were maintained in mTeSR™1 medium (StemCell). The ACM differentiation was performed by promoting RA signaling using commercial atrial cardiomyocyte differentiation kit (Stemcell) based on the manufacturer's instructions (Jiang *et al.*, 2021; Zhang *et al.*, 2021). The cells were cultured in a humidified atmosphere of 5% CO2 at 37 °C.
The HL-1 cell line and hiPSC-ACM were transfected either with an adenovirus vector carrying the *PRKAG2*-R302Q mutation or a negative control (GeneChem).

6. Western blot

Total proteins from the patients' left atrial appendage samples were collected using a total protein extraction kit (KeyGen BioTECH, KGP250). The protein concentration was quantified using a Pierce™ BCA protein assay kit (Thermo Fisher, 23225). The proteins were separated by SDS–PAGE and transferred onto PVDF membranes. The membrane was blocked with 5% BSA and incubated with the following primary antibodies (4 °C, overnight): phospho-AMPKα (CST, 2535), AMPKα (CST, 5831), glycogen synthase 1/GYS1 (Abcam, ab40810), GLUT4 (Affinity, AF5386), and GAPDH (Proteintech, 10494-1-AP). An HRP-conjugated secondary antibody (CST) was added to the membrane, which was left for 2 h. After the addition of chemiluminescent reagents (Thermo Fisher Scientific, 17295), bands were detected using a Molecular Imager Chemi-Doc™ XRS+ Imaging System (Bio–Rad) and quantified using Image Lab™ Software (Bio–Rad).

7. ELISA

A total of 6×10^5 iPSC-ACMs or 1×10^6 HL-1 cells were prepared for ELISA. After 48

h of transfection, 200 μM adenosine 5'-monophosphate monohydrate (AMP) (Selleck, S5284) was added to the cells and left for 20 min. The cells were digested and lysed by ultrasonication for protein extraction. The phospho-AMPKα concentrations were determined using ELISA kits (MEIMIAN, MM-44247M1). The absorbance at 450 nm was read within 15 min of the addition of a stop solution.

8. Histological evaluation

Left atrial appendage tissues from the proband and controls were fixed in 4% formalin and embedded in paraffin. The 4-μm sections were stained using the hematoxylin and eosin (H&E), Masson, and periodic acid–Schiff (PAS) techniques. In addition, HL-1 cells were preserved in formalin at 48 h post-transfection for PAS analysis.

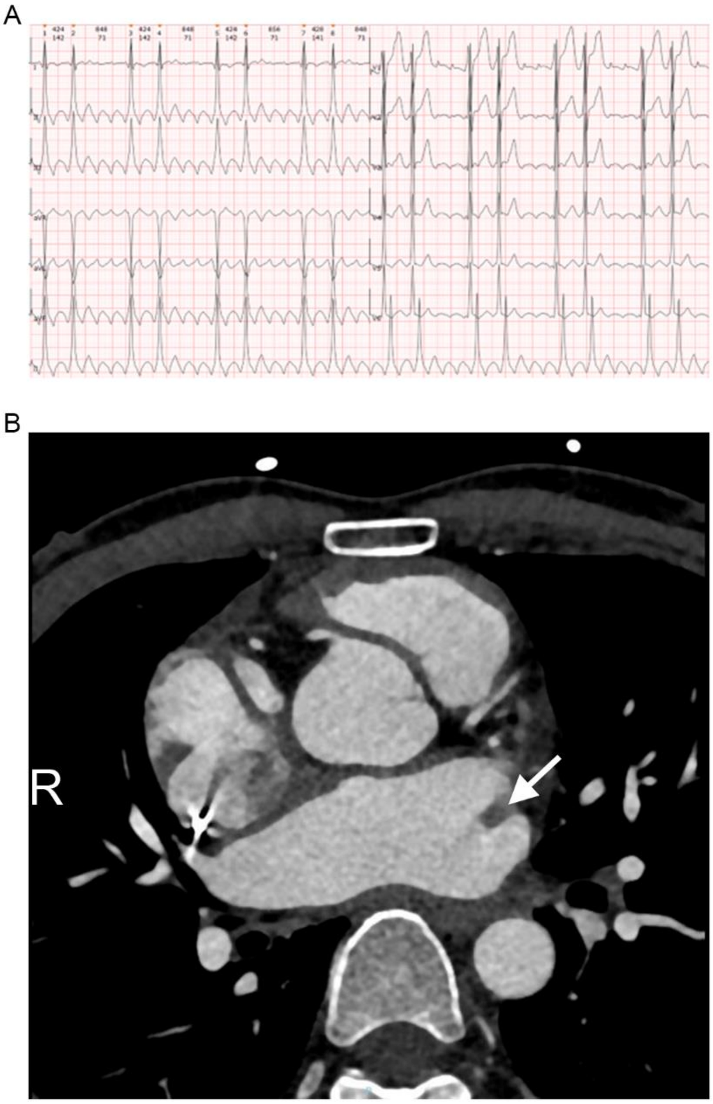

Supplementary Figure 1. (A) Electrocardiogram (ECG) of the proband upon the diagnosis of atrial flutter. **(B)** Cardiac CT-scan of the proband after 4-month anticoagulant therapy.

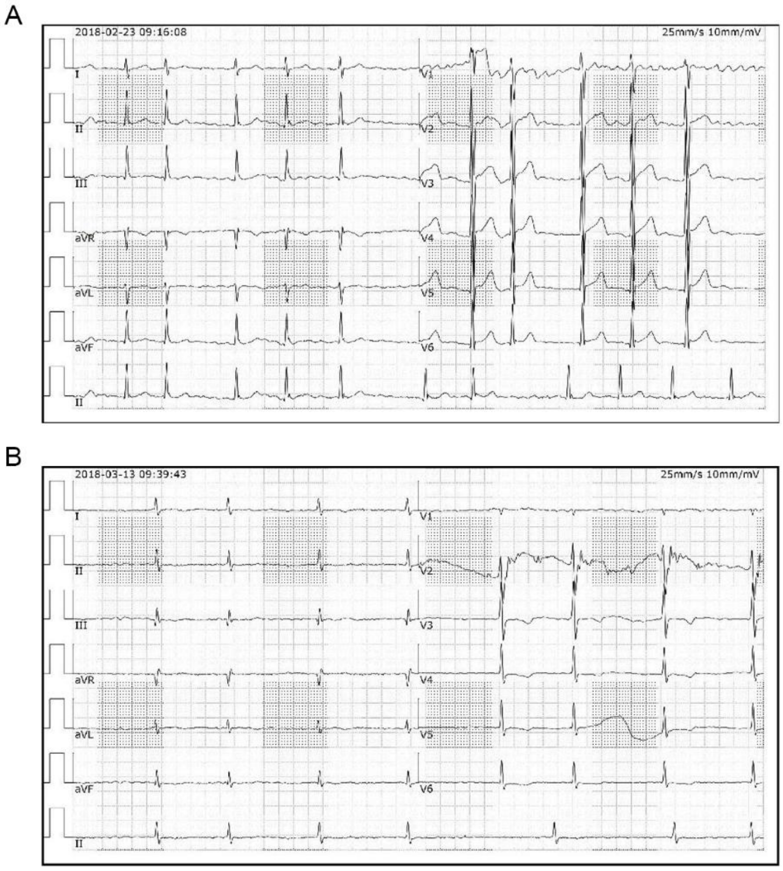

Supplementary Figure 2. Electrocardiogram of the control 1 **(A)** and control 2 **(B)** patient.

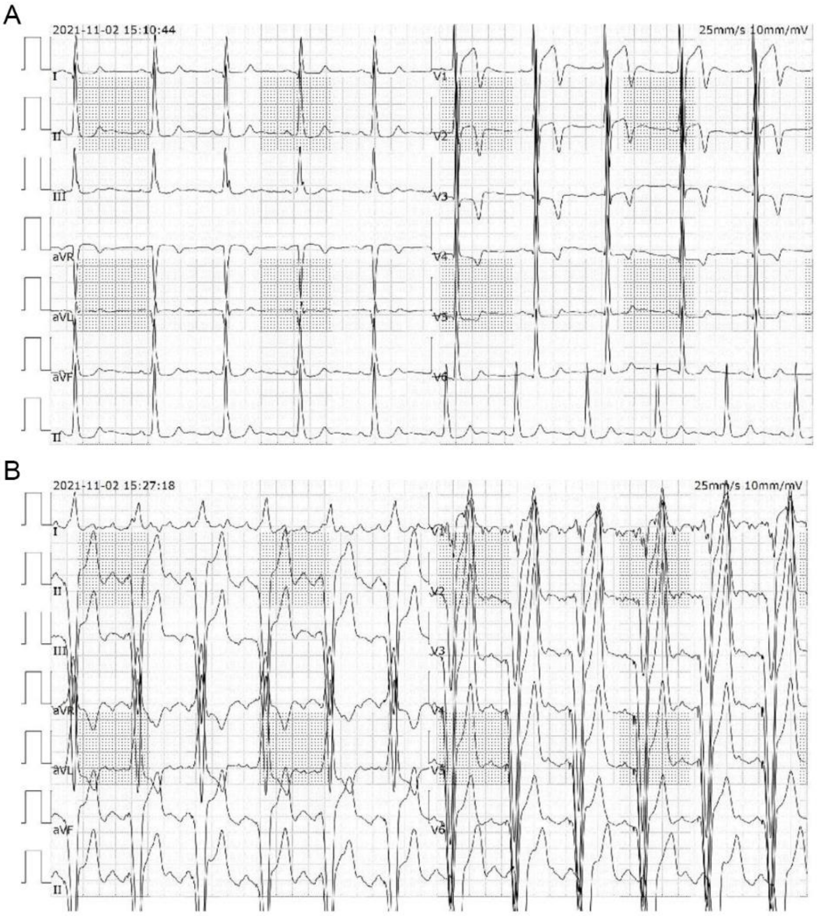

Supplementary Figure 3. Electrocardiogram of the proband **(A)** and II-3 **(B)** at the three-year follow-up.

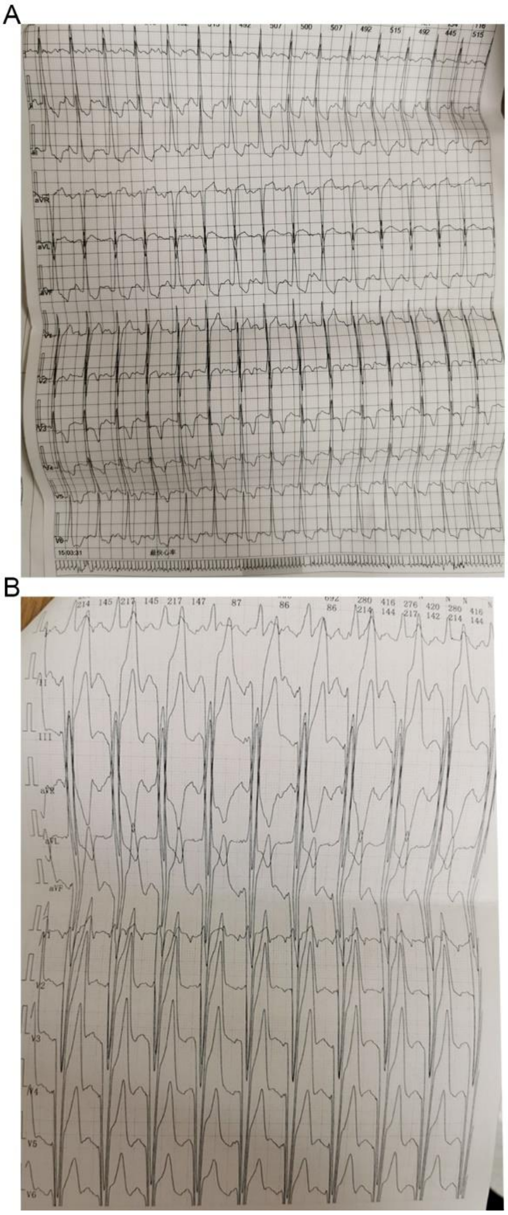

Supplementary Figure 4. 24-h Holter-ECG monitoring of the proband **(A)** and II-3 **(B)** at the three-year follow-up.

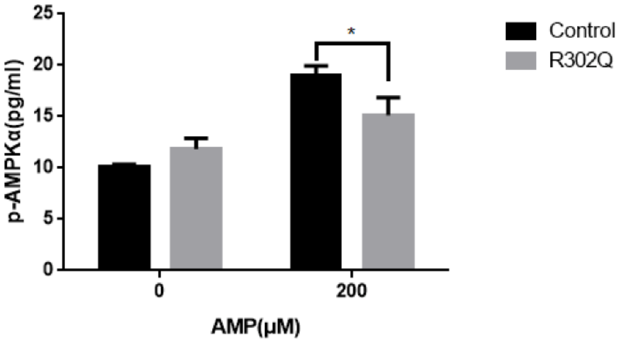

Supplementary Figure 5. The level of p-AMPKα expression obtained from hiPSC-ACMs was determined by ELISA (n=3). The data were analyzed by the Student's t-test (n=3, $^{*}p<0.05$).

Supplementary Table 1. Echocardiographic data of the proband, III-1, II-3, control 1 and 2 patient.

	Proband	III-1	II-3	Control 1	Control 2
Aod (mm)	27	26	30	28	31
LAD (mm)	39	27	32	73	54
LVDd (mm)	40	40	49	45	50
LVDs (mm)	25	25	32	30	33
IVS (mm)	18	8	15	10	10
LVPW (mm)	10	8	11	10	10
RAD (mm)	38	30	34	43	37
RVDd (mm)	32	28	32	46	33
FS (%)	37.5	37.5	34.7	33	34
EF (%)	68.1	68.1	63.7	62.1	62.7

Abbreviation: Aod, Aorta diameter; LAD, left atrial diameter; LVDd, left ventricular end-diastolic diameter; LVDs, left ventricular end-systolic diameter; IVS, interventricular septum; LVPW, left ventricular posterior wall; RAD, right atrial diameter; RVDd, right ventricular end-diastolic diameter; FS, fractional shortening; EF, ejection fraction.

Supplementary Table 2. Echocardiographic data of the proband and II-3 at the three-year follow-up.

	Proband	II-3
Aod (mm)	26	30
LAD (mm)	37	40
LVDd (mm)	43	48
LVDs (mm)	28	30
IVS (mm)	13	18
LVPW (mm)	11	14
RAD (mm)	35	39
RVDd (mm)	38	39
FS (%)	34.9	37.5
EF (%)	64.4	67.4

Abbreviation: Aod, Aorta diameter; LAD, left atrial diameter; LVDd, left ventricular end-diastolic diameter; LVDs, left ventricular end-systolic diameter; IVS, interventricular septum; LVPW, left ventricular posterior wall; RAD, right atrial diameter; RVDd, right ventricular end-diastolic diameter; FS, fractional shortening; EF, ejection fraction.

[1] X. Jiang, H. Cheng, J. Huang, C. Cui, Y. Zhu, Y. Lin, et al. (2021). Construction of chamber-specific engineered cardiac tissues in vitro with human iPSC-derived cardiomyocytes and human foreskin fibroblasts. *Journal of bioscience and bioengineering*. 132, 198-205. doi:10.1016/j.jbiosc.2021.04.012

[2] Y. Zhang, Y. Zhu, Y. Lin, H. Liu, H. Chen, W. Ju, et al. (2021). Establishment of an iPSC line (JSPHi001-A) from a patient with familial dilated cardiomyopathy and atrial fibrillation caused by LMNA missense mutation (c.1003C > T). *Stem Cell Res*. 53, 102349. doi:10.1016/j.scr.2021.102349
